# New method of video‐assisted vaginoscopy in Nellore heifers

**DOI:** 10.1002/vms3.1232

**Published:** 2023-09-07

**Authors:** Gabriela Jaques Rodrigues, Bruno Moura Monteiro, Rinaldo Batista Viana, Aluizio Otavio Almeida da Silva, Francisco Décio de Oliveira Monteiro, Pedro Paulo Maia Teixeira

**Affiliations:** ^1^ Instituto of Veterinary Medicine Federal University of Pará (UFPA), Campus Castanhal Castanhal Pará Brazil; ^2^ Institute of Health and Animal Production Federal Rural University of the Amazon Belém Pará Brazil; ^3^ Federal Institute of Tocantins (IFTO), Campus Araguatins Araguatins Tocantins Brazil

**Keywords:** intravaginal inspection, vaginal endoscopy, vaginal exam, vaginoscopy in cows, videodiagnosis

## Abstract

Vaginoscopy allows an intravaginal endoscopic evaluation and can help in the diagnosis of female bovine genital tract disorders. The aim of this study is to validate a new method of gynaecological examination using a Scope VOR&GDI videovaginoscope. Twenty‐six heifers were used, divided into 2 groups with 13 animals, control group (GC) and videovaginoscopic group (GV). In the CG, vaginoscopy was performed with a vaginal speculum and in the GV with a Scope VOR&GDI videovaginoscope. All heifers underwent vaginoscopy on day 0 (D0), and 9 days later, on day 9 (D9). Vaginoscopy provided adequate intravaginal inspection. In the CG, 23% (3/13) of the heifers showed discomfort during the test. In GV, vaginal inspection was better due to better image quality. The videovaginoscope is the most effective equipment for carrying out the vaginoscopy procedure in Nellore heifers, as it produces sharper and clearer images and can help in the diagnostic and therapeutic routine of veterinarians.

## Graphical abstract

Vaginoscopy was performed on 2 groups of 13 heifers, a control group using a vaginal speculum and a test group using a new Scope VOR&GDI instrument. The new instrument showed better efficiency for identifying intravaginal structures and characteristics with better image quality and comfort for the examiner and patient.

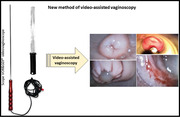

1

The vaginoscopy is a procedure that allows detailed endoscopic evaluation of the vaginal walls, fornices and exocervix. The procedure is simple and can aid in the diagnosis of female bovine genital tract disorders. Examination with a speculum usually allows inspection of the caudal part of the vagina and causes discomfort in some animals. Vaginal endoscopy is considered a superior diagnostic procedure as it allows evaluation of the entire vagina (Di Spiezio Sardo et al., 2016; Levy, 2016).

Vaginal endoscopy allows you to examine the nature and extent of disease in the vagina and, in some cases, provides treatment options. The technological resources used in video diagnostic methods are being increasingly applied. Videovaginoscopy in bitches and vaginoscopy in sheep with laparoscopic surgical portal are advances that allow a better diagnostic exploration in the intravaginal route (Easley et al., 2017; Levy, 2016).

The examination with a vaginal speculum provides a smaller field of view when compared to vaginal endoscopy, and this may induce or preclude a more assertive diagnosis. The endoscopic resource is a modern solution, as endoscopic equipment can generate sharper and clearer images, improving the sensitivity of the diagnosis. Vaginal endoscopy is used both as a diagnostic tool, for a complete reproductive evaluation, and for intrauterine and therapeutic procedures (Maenhoudt and Santos, 2021).

The use of videovaginoscopy in cattle has not been reported in the literature, and therefore, we believe that this technique can be successfully used in this species. Thus, the aim of this study is to validate a new method of gynaecological examination using a Scope VOR&GDI videovaginoscope.

This study was carried out in accordance with the recommendations of the Conselho Nacional de Controle de Experimentação Animal do Brasil (CONCEA). This research was approved by the Ethics and Animal Welfare Committee of the Federal University of Pará (CEUA/UFPA), protocol no 8762300120. The procedures did not cause pain or suffering to the animals and were performed under the supervision of CEUA/UFPA.

The experiment was conducted on a farm in the municipality of Peixe‐Boi, in the state of Pará/Brazil. Twenty‐six Nellore bovine females, heifers, with an average age of 2 years and an average body weight of 280 kg were selected. The heifers were examined in order to rule out those that were sick or unfit for reproduction. During the clinical examination, vital and clinical signs were evaluated through inspection, palpation, percussion and auscultation, considering the general condition of the females. Examinations of the mucous membranes and lymph nodes were performed through inspection and palpation, and the specific examination of the reproductive system by rectal palpation and ultrasound.

The 26 heifers underwent a hormonal protocol to induce puberty for 12 days based on progesterone (DIB) and oestradiol (ECP). Subsequently, they were submitted to oestrus synchronization with a hormonal protocol based on progestogens, oestrogens and equine chorionic gonadotropin (ECG‐Novonorm), followed by fixed‐time artificial insemination.

All heifers underwent vaginoscopy on the first day of hormone application for oestrus synchronization for TAI, on day 0 (D0), and 9 days later, on day 9 (D9), randomly and by the same examiner. The 26 heifers were divided into two groups, control group (CG) and videovaginoscopic group (GV), 13 heifers in each group. In the CG, vaginoscopy was performed with a vaginal speculum and in the GV with a Scope VOR&GDI videovaginoscope (Figure 1).

**FIGURE 1:**
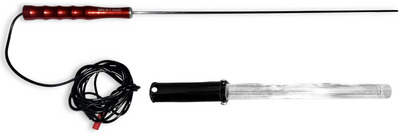
New equipment used in vaginoscopy exams, Scope VOR&GDI vaginoscope.

In the CG, a common stainless steel vaginal speculum with an external flashlight was used. In the GV, a VOR&GDI videovaginoscope scope composed of two parts was used, a tubular acrylic speculum measuring 3.5 cm in diameter and 32 cm in length, washable, light and transparent, allowing good visualization and intravaginal illumination, and an optical scope 160 K with micro camera on the rod. The speculum also has an entry for a biopsy needle, a pipette for uterine lavage and a semen applicator (Figure 1).

During the vaginoscopy, the vaginal region up to the entrance of the cervix was visualized and photographed. In the CG, the images were captured using a cell phone camera, model IPhone 11. In the GV, the images were captured by the VOR&GDI videovaginoscope equipment camera. The images produced were analysed blindly by three evaluators proficient in gynaecological examination of bovines.

The evaluation consisted of identifying intravaginal structures and characteristics such as mucosal colour, mucosal moisture, shape of the entrance to the cervix and degree of opening of the cervical canal. Visualization scores were determined for each analysed variable. A numbering was established for each score: (1) score 1 (clear and clear visualization); (2) score 2 (visualization unclear); (3) score 3 (difficult visualization) and (4) score 4 (no visualization).

Visualization scores (1–4) were statistically analysed in the Minitab program (Minitab Inc., Copyright C 2005, version 14.20). Data were checked for normality using the Kolmogorov–Smirnov test. In the samples that showed normality, the *t* test was applied. For the analysed parameters, a significance level of *p* < 0.005 was considered.

Vaginoscopy performed in all heifers provided adequate intravaginal inspection in both groups, GC and GV. In the GV, no behavioural alteration was identified that would indicate discomfort during the vaginal introduction of the VOR&GDI videovaginoscope equipment. In the CG, 23% (3/13) of the heifers showed discomfort during the introduction of the vaginal speculum.

The best intravaginal inspection was obtained in GV due to the better quality of the images. Table 1 presents the data related to the analysis of the variables. There was no statistical difference between the groups, only in the variable colour of the vaginal mucosa (*p* = 0.09).

**TABLE 1:** Average and standard error of visualization scores of intravaginal features assessed by vaginoscopy in GC and videovaginoscopic group (GV).

	Average		Standard error		
Intravaginal features	GV	GC	GV	GC	*p*
Coloration of the vaginal mucosa	1.8462^a^	2.0513^a^	0.0732	0.0964	0.090
Moisture of the vaginal mucosa	1.9872^a^	2.3846^b^	0.0763	0.0898	0.001
Shape of the entrance to the cervix	1.9487^a^	2.5128^b^	0.0873	0.0995	0.002
Opening of the cervix	1.7692^a^	2.3460^b^	0.0773	0.109	0.001

*Note*: Average with letters different from each other differs statistically.

It is possible to observe the difference in the images of the GV (Figure 2a) and the CG (Figure 2b), in addition to special findings in the GV such as the presence of haemorrhagic points in the epithelium (Figure 2c). It was also possible to visualize a cervical plug formed by mucus at the entrance to the cervix (Figure 2d). In the CG, it was not possible to clearly visualize the details of the vaginal epithelium, one animal in this group presented bleeding and discomfort during the introduction of the vaginoscope (Figure 2e,f).

**FIGURE 2:**
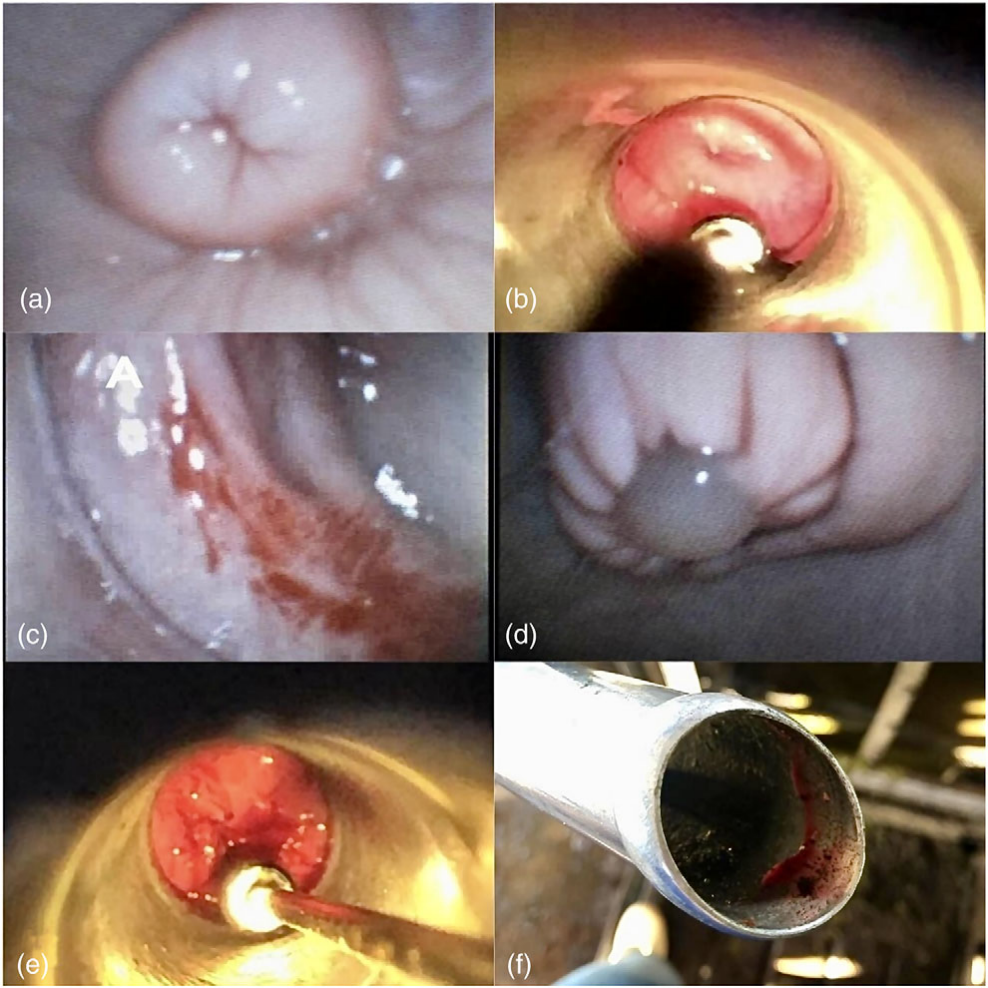
a – cervix visualized with the VOR&GDI videovaginoscope; b – cervix visualized with a standard vaginoscope; c – bleeding points on the vaginal mucosa; d – mucous plug at the entrance of the cervical canal; e – bleeding mucosa after vaginoscope port; f – equipment with blood after removal of the vaginal canal.

In the histogram, it is possible to observe a normal distribution of the visualization scores by group (Figures 3 and 4). In the CG, the variable ‘Coloration of the vaginal mucosa’ showed an equivalent distribution of scores 1–3, whereas in the GV, there was a higher frequency of score 2 (Figure 3a). In the CG, the variable ‘Humidity of the vaginal mucosa’ presented a higher frequency of score 3, whereas in the GV, score 2 was more frequent (Figure 3b). As for viewing the format of the entrance and opening of the cervix, there was a higher frequency of score 2 in both groups (Figure 4a,b).

**FIGURE 3:**
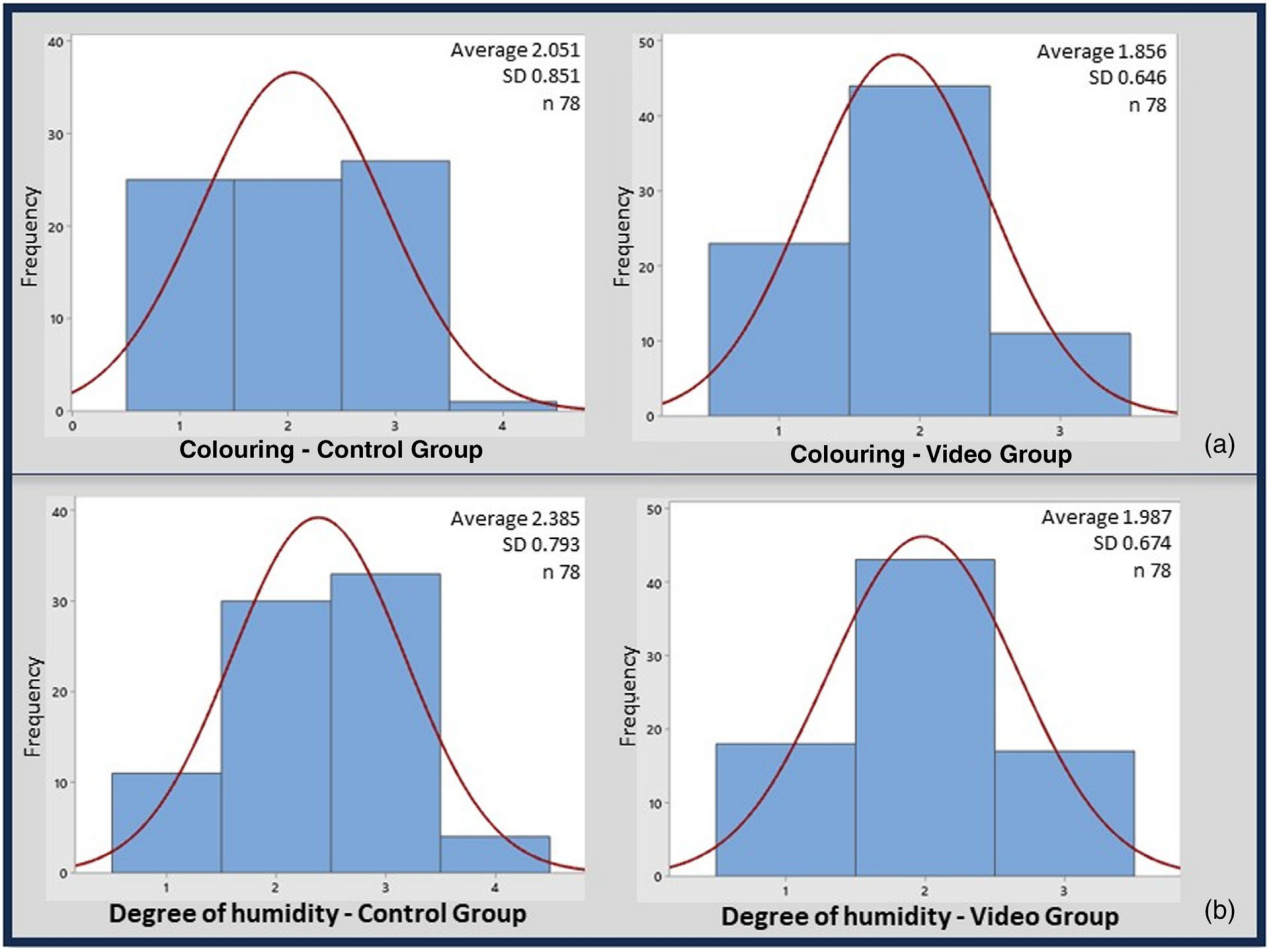
Histograms with normal curve of distribution of visualization scores: a – colouring of mucosa, b – moisture level of the mucosa.

**FIGURE 4:**
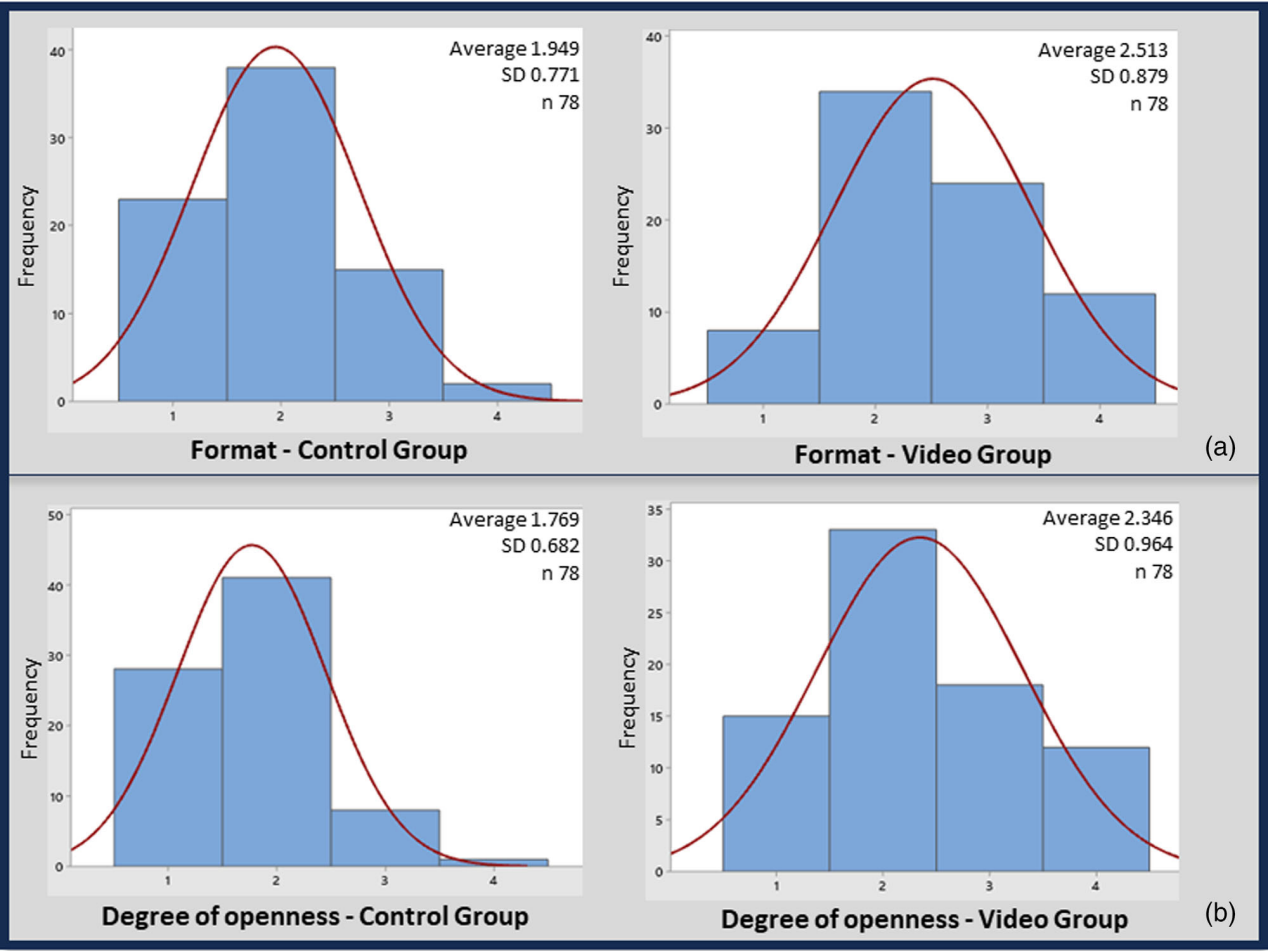
Histograms with normal curve of distribution of visualization scores: a – shape of the vaginal portion of the cervix, b – degree of opening of the cervical canal.

The results showed that to evaluate intravaginal structures, the Scope VOR&GDI videovaginoscopy equipment was more effective, with better visualization scores. The exams were easily performed on the GV, as the equipment is more anatomical and suitable for introduction into the vaginal canal. Thus, the equipment can provide a quick and non‐traumatic examination in a complete reproductive evaluation and intrauterine procedures in cattle (Marques et al., 2011).

The vaginoscopy is used in cattle, horses, dogs and sheep to aid in the therapy and diagnosis of some diseases such as endometritis, metritis and vaginitis (Additional and complementary results can be consulted in the supplementary files contained in this manuscript.). (Sheldon et al., 2009; Gobikrushanth et al., 2016). In this exam, it is possible to directly observe the intravaginal structures in a minimally invasive way, especially when associated with the video diagnostic resource (Easley et al., 2017, Tison et al., 2017).

In most dairy cattle setups, heifers are not subject to vaginoscopy unless there is an obvious reason to do so. Vaginoscopy was performed in heifers in order to test the new method, in a precursory way, validating the method that can be tested and used in post‐partum cows. Heifers induced to heat allowed a better visualization of the vaginal mucosa due to hormonal action.

Vaginoscopy is more sensitive than vaginal cytology for diagnosing clinical and subclinical post‐partum endometritis. It is an important resource for diagnosing these conditions, especially if rectal palpation is the only diagnostic method, but the technique has limitations. The visual evaluation of the images by the common vaginoscope, illuminated by an external flashlight, was more difficult when compared to the new model of video‐assisted vaginoscopy (Barlund et al., 2008).

Vaginoscopy performed using the conventional method, illuminated with an external flashlight, showed lower sensitivity and specificity, demonstrating that this method can complicate the visual assessment with an increased tendency to error (Leutert et al., 2012). We believe that this is influenced by the smaller visual field with limited lighting. The images generated by the conventional vaginoscope showed low sharpness and difficult visualization.

In other species, such as dogs and sheep, vaginoscopy is also an effective tool for reproductive management, identifying oestrus stages, predicting other reproductive stages and facilitating transcervical insemination (Levy, 2016; Tison, et al., 2017). However, in these animals, conventional vaginoscopy can result in discomfort, being inappropriate for smaller patients (Di Spiezio Sardo et al., 2016). In this case, rigid endoscopes are used to perform tests and insemination, as there is no vaginoscopy equipment with a video camera.

In cows and buffaloes, there are no reports of the use of rigid endoscopes for vaginoscopy, for this reason the videovaginoscope brought the innovation of video‐assisted vaginoscopy that can help veterinarians in their diagnostic and therapeutic routine. The use of videovaginoscopy is a new tool that can be used in dairy cattle because it offers less discomfort and less trauma to cows and will be readily accepted by cattle breeders (Leutert et al., 2012; Helfrich et al., 2020).

The videovaginoscope is the most effective equipment for performing the procedure of vaginoscopy, as it produces sharper and clearer images and can help in the diagnostic and therapeutic routine of veterinarians. Future work may investigate the feasibility of vaginoscopy using our Scope VOR&GDI videovaginoscope in post‐partum cows, or even in other species, to assess the endoscopic quality of the vaginal mucosa and its morphology.

## AUTHOR CONTRIBUTIONS

*Conceptualization; methodology; project administration and supervision*: Gabriela Jaques Rodrigues, Aluizio Otavio Almeida da Silva and Pedro Paulo Maia Teixeira. *Data curation; formal analysis and investigation and resources*: Francisco Décio de Oliveira Monteiro, Bruno Moura Monteiro, Rinaldo Batista Viana and Pedro Paulo Maia Teixeira. *Visualization, writing – original draft and writing – review and editing*: Gabriela Jaques Rodrigues, Francisco Décio de Oliveira Monteiro and Pedro Paulo Maia Teixeira.

## CONFLICT OF INTEREST STATEMENT

The authors declare no conflicts of interest.

## FUNDING INFORMATION

None

### ETHICS STATEMENT

This study was carried out in accordance with the recommendations of the Conselho Nacional de Controle de Experimentação do Brasil (CONCEA). This research was approved by the Ethics and Animal Welfare Committee of the Federal University of Pará (CEUA/UFPA), protocol no 8762300120.[Supplementary-material vms31232-supitem-0001]

### PEER REVIEW

The peer review history for this article is available at https://publons.com/publon/10.1002/vms3.1232.

## Supporting information

Supporting Information

Supporting Information

Supporting Information

## Data Availability

No.

## References

[vms31232-bib-0001] Barlund, C. S. , Carruthers, T. D. , Waldner, C. L. , & Palmer, C. W. (2008). A comparison of diagnostic techniques for postpartum endometritis in dairy cattle. Theriogenology, 69, 714–723. 10.1016/j.theriogenology.2007.12.005 18242670

[vms31232-bib-0002] Di Spiezio Sardo, A. , Zizolfi, B. , Calagna, G. , Florio, P. , Nappi, C. , & Di Carlo, C. (2016). Vaginohysteroscopy for the diagnosis and treatment of vaginal lesions. International Journal of Gynecology & Obstetrics, 133, 146–151. 10.1016/j.ijgo.2015.09.018 26892691

[vms31232-bib-0003] Easley, J. , Shasa, D. , & Hackett, E. (2017). Vaginoscopy in ewes utilizing a laparoscopic surgical port device. Journal of Veterinary Medicine, 2017, 1–4. 10.1155/2017/740437 PMC5613375 29138758

[vms31232-bib-0004] Gobikrushanth, M. , Salehi, R. , Ambrose, D. J. , & Colazo, M. G. (2016). Categorization of endometritis and its association with ovarian follicular growth and ovulation, reproductive performance, dry matter intake, and milk yield in dairy cattle. Theriogenology, 86, 1842–1849. 10.1016/j.theriogenology.2016.06.003 27395084

[vms31232-bib-0005] Helfrich, A. L. , Reichenbach, H. , Meyerholz, M. M. , Schoon, H. , Arnold, G. J. , Fröhlich, T. , Weber, F. , & Zerbe, H. (2020). Novel sampling procedure to characterize bovine subclinical endometritis by uterine secretions and tissue. Theriogenology, 141, 186–196. 10.1016/j.theriogenology.2019.09.016 31557616

[vms31232-bib-0006] Leutert, C. , von Krueger, X. , Plöntzke, J. , & Heuwieser, W. (2012). Evaluation of vaginoscopy for the diagnosis of clinical endometritis in dairy cows. Journal of Dairy Science, 95, 206–212. 10.3168/jds.2011-4603 22192199

[vms31232-bib-0007] Levy, X. (2016). Videovaginoscopy of the canine vagina. Reproduction in Domestic Animals, 51, 31–36. 10.1111/rda.12785 27670938

[vms31232-bib-0008] Maenhoudt, C. , & Santos, N. R. (2021). Vaginal endoscopy in the dog. Veterinary Endoscopy for the Small Animal Practitioner, 363–381. 10.1002/9781119155904.ch7

[vms31232-bib-0009] Marques, A., Jr , Martins, T. , & Borges, Á. (2011). Diagnostic approach and treatment of uterine infection in cows. Rev. Bras. Animation Reproduction, 35, 293–298.

[vms31232-bib-0010] Sheldon, I. M. , Cronin, J. , Goetze, L. , Donofrio, G. , & Joachin, H. (2009). Defining postpartum uterine disease and the mechanisms of infection and immunity in the female reproductive tract in cattle. Biology of Reproduction, 81, 1025–1032. 19439727 10.1095/biolreprod.109.077370 PMC2784443

[vms31232-bib-0011] Tison, N. , Bouchard, E. , DesCôteaux, L. , & Lefebvre, R. C. (2017). Effectiveness of intrauterine treatment with cephapirin in dairy cows with purulent vaginal discharge. Theriogenology, 89, 305–317. 10.1016/j.theriogenology.2016.09.007 28043367

